# Paleo-diatom composition from Santa Barbara Basin deep-sea sediments: a comparison of *18S-V9* and *diat-rbcL* metabarcoding vs shotgun metagenomics

**DOI:** 10.1038/s43705-021-00070-8

**Published:** 2021-11-09

**Authors:** Linda Armbrecht, Raphael Eisenhofer, José Utge, Elizabeth C. Sibert, Fabio Rocha, Ryan Ward, Juan José Pierella Karlusich, Leila Tirichine, Richard Norris, Mindi Summers, Chris Bowler

**Affiliations:** 1 Institute for Marine and Antarctic Studies (IMAS), Ecology & Biodiversity Centre, University of Tasmania, Battery Point, TAS 7004 Australia; 2 Australian Centre for Ancient DNA, School of Biological Sciences, Faculty of Sciences, The University of Adelaide, Adelaide, SA 5005 Australia; 3 Institut de Biologie de l’Ecole Normale Supérieure (IBENS), Ecole Normale Supérieure, CNRS, INSERM, Université PSL, 75005 Paris, France; 4 Australian Research Council Centre of Excellence for Australian Biodiversity and Heritage, The University of Adelaide, Adelaide, SA 5005 Australia; 5 UMR 7206, Muséum National d’Histoire Naturelle, CNRS, Université Paris Diderot, 75016 Paris, France; 6 Department of Earth and Planetary Sciences, Yale University, New Haven, CT 06511 USA; 7 Yale Institute for Biospheric Studies, Yale University, New Haven, CT 06511 USA; 8 Université de Nantes, CNRS, UFIP, UMR 6286, F-44000 Nantes, France; 9 GRD, Scripps Institution of Oceanography, UC San Diego, La Jolla, CA 92093-0244 USA; 10 Department of Biological Sciences, University of Calgary, Calgary, AB T2N 1N4 Canada

**Keywords:** Metagenomics, Biodiversity, Molecular ecology

## Abstract

Sedimentary ancient DNA (*sed*aDNA) analyses are increasingly used to reconstruct marine ecosystems. The majority of marine *sed*aDNA studies use a metabarcoding approach (extraction and analysis of specific DNA fragments of a defined length), targeting short taxonomic marker genes. Promising examples are *18S-V9 rRNA* (~121–130 base pairs, bp) and *diat-rbcL* (76 bp), targeting eukaryotes and diatoms, respectively. However, it remains unknown how *18S-V9* and *diat-rbcL* derived compositional profiles compare to metagenomic shotgun data, the preferred method for ancient DNA analyses as amplification biases are minimised. We extracted DNA from five Santa Barbara Basin sediment samples (up to ~11 000 years old) and applied both a metabarcoding (*18S-V9 rRNA*, *diat-rbcL*) and a metagenomic shotgun approach to (i) compare eukaryote, especially diatom, composition, and (ii) assess sequence length and database related biases. Eukaryote composition differed considerably between shotgun and metabarcoding data, which was related to differences in read lengths (~112 and ~161 bp, respectively), and overamplification of short reads in metabarcoding data. Diatom composition was influenced by reference bias that was exacerbated in metabarcoding data and characterised by increased representation of *Chaetoceros*, *Thalassiosira* and *Pseudo-nitzschia*. Our results are relevant to *sed*aDNA studies aiming to accurately characterise paleo-ecosystems from either metabarcoding or metagenomic data.

## Introduction

Sedimentary ancient DNA (*sed*aDNA) analyses have become increasingly applied to the sub-seafloor for the reconstruction of marine ecosystems. Using *sed*aDNA, taxa across all three domains of life (archaea, bacteria, eukaryota) have been detected, including non-fossilising species (e.g., [1, 2]). The latter shows the enormous potential of *sed*aDNA techniques to go beyond standard environmental proxies and facilitate the reconstruction of paleo-ecosystems across the entire marine food web, rather than the small proportion of marine biodiversity detectable from fossils alone.

Amongst the most popular study targets are eukaryotes, especially microscopic phytoplankton, key environmental indicators whose compositional changes reflect changes in past ocean conditions and climate [3–5]. Furthermore, DNA sequences from photosynthetic organisms in deep-sea sediments are more likely of ancient origin than from living contaminants because these organisms require light for their survival. Particularly important are the diatoms, which are responsible for ~20% of annual global net primary production [6, 7]. Diatom microfossils have been characterised extensively in sediment cores to predict past ecosystems (e.g., [8, 9]). However, studying marine eukaryotes by means of *sed*aDNA has remained complicated as only minuscule amounts of their DNA are preserved in the sub-seafloor (~1.5% of total DNA is of eukaryote origin when using the small subunit ribosomal RNA (SSU) taxonomic marker gene as a ref. [10]).

Most *sed*aDNA studies have used a metabarcoding approach to maximise the genetic signal of eukaryotes. Metabarcoding targets a specific DNA region, such as a taxonomic marker gene, enabling the identification of different species within a sample [11]. These genetic markers are amplified using primers (short sequences matching the start and end of the target gene) in a polymerase chain reaction (PCR) and subsequently sequenced. A frequently used marker gene for marine eukaryotes is the SSU rRNA (18S rRNA) or shorter regions within this gene, such as *18S-V1, 18S-V3, 18S-V7, 18S-V9* [3, 5, 12–14]. The hypervariable gene region *18S-V9* is particularly well characterised as a result of global ocean sampling programs focusing on the study of marine eukaryotes [15–18], providing an extensive number of modern references (e.g., summarised in the protist ribosomal database, PR^2^ [19]). Furthermore, *18S-V9* is quite short, ranging from 87 to 186 bp (average 121 bp, most sequences ~130 bp [15]).

There are a few reasons, however, why metabarcoding is problematic when applied to *sed*aDNA. Ancient DNA is typically very fragmented and damaged, often preventing PCR primers from binding to it [20]. Also, target sequences are usually longer than the short ancient DNA fragments (<100 bp [21, 22]), resulting in preferential amplification of better preserved DNA molecules—a bias that can be further enhanced by the random amplification of DNA fragments in the first few PCR cycles (PCR bias, especially when many cycles are applied [23–25]). These issues can significantly distort the results, with the final data being heavily biased towards well-preserved sequences, possibly from contaminant taxa. Similarly, previous paleo-microbiome research using the bacterial taxonomic marker gene 16S rRNA has shown that extensive length variations in the *16S-V3* region are a major cause of differential amplification resulting in taxonomic bias in ancient microbiome reconstructions, preventing them from being accurate [26].

A preferred technique in *sed*aDNA research is to use a metagenomics approach that relies on the extraction and amplification of the ‘total’ DNA (‘shotgun’ approach), facilitating the investigation of potentially all species in a sample and independent of DNA fragment size [11, 26]. Amongst the first metagenomics *sed*aDNA studies in marine environments were investigations from the Arabian Sea (e.g., [4, 27]). However, if the DNA of the target organisms is rare compared to the total extracted DNA (as for eukaryotes in *sed*aDNA), very deep sequencing (achieving a high number of reads) is required to recover sufficient genetic information and perform meaningful statistical analyses. Often, the total pool of metagenomic shotgun data is screened for the occurrence of a taxonomic marker gene, such as the SSU and large subunit ribosomal RNA (LSU) whereby only a fraction of the data is kept for downstream analyses, reducing cost-effectiveness.

Recent metagenomics studies using sediments from Australia and Antarctica have shown that marine *sed*aDNA can be very short, with most sequences being ~70 and ~40–50 bp, respectively [10, 28]. This is even shorter than the minimum fragment length of the *18S-V9* region (87 bp [15]), suggesting that the application of *18S-V9* metabarcoding might lead to similar skewed eukaryote composition reconstructions as is the case for paleo-prokaryotes using *16S-V3* [26]. A more suitable target gene region might be one that is closer to the typical ~40 - 70 bp *sed*aDNA fragment size, such as the diatom-specific *diat-rbcL* gene region, a 76 bp region within the gene encoding the large subunit of the enzyme ribulose-1,5-bisphosphate carboxylase-oxygenase (*rbcL*) [29]. The *rbcL* gene is relatively conserved (more than the SSU) and thus discriminates well between phytoplankton taxa at species level [30, 31]. *Diat-rbcL* has been used previously to investigate diatom composition in tropical and Arctic lakes, as well as in Arctic marine sediments [29, 32, 33].

Here, we provide the first comparison of metagenomic and metabarcoding derived eukaryote *sed*aDNA data from the Santa Barbara Basin. We selected both *18S-V9* and *diat-rbcL* for our metabarcoding approach, testing whether they capture a similar breadth of eukaryote and diatom diversity to shotgun data. We investigated paleo-eukaryote composition and taxon-specific *sed*aDNA fragment lengths, and whether the latter, and/or potential reference biases, impacted the taxonomic profiles.

## Methods

### Sediment core sampling

**Fig. 1:**
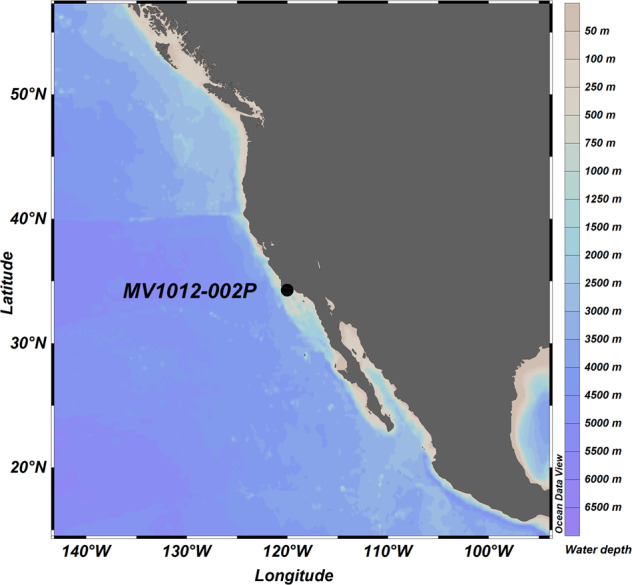
Map of MV1012-002P coring site in the Santa Barbara Basin. The exact coordinates are 34.288°N, 120.036°W. ODP Site 893 A is <100 m away from MV1012-002 thus not depicted here. Map created in ODV (Schlitzer, R., Ocean Data View, https://odv.awi.de, 2018).

An 18 m long Jumbo Piston Core (MV1012-002P) was collected at ~576.5 m water depth in the Santa Barbara Basin off California, USA, during the CalEchoes MV1012 expedition (28 September 2010, *R/V Melville*) (Fig. 1). The core was cut into 1.5 m sections, each was capped, vacuum-bagged with nitrogen gas and a commercial oxygen-absorber, sealed and transported to the Scripps Institution of Oceanography Geological Collection (SIO-GC) for storage (4 °C). In 2017, the bottom 25 cm of five sections (Sections 1, 3, 5, 8, 11, each 10.16 cm diameter in PVC liner) were cut off using sterile tools while wearing gloves and face masks to minimize contamination. The 25 cm sections were re-bagged/-sealed, kept at 4 °C and subsampled for *sed*aDNA analysis at the paleogenomics facilities of the Musée de l’Homme, Paris, France, in 2018.

The bag containing Section 1 (youngest) was slightly damaged, thus was prioritised for *sed*aDNA sampling to minimise rapid *sed*aDNA degradation. Afterwards, we worked from bottom to top sections. We decontaminated the lab (Surfa’Safe Premium, ANIOS, France), placed a fresh bench cloth, and changed gloves between cutting and sampling of each section. A Dremel EZ cutter fitted with a SpeedClic adapter and a 38 mm metal cutting disc (replaced after each section) was used for splitting. We scooped ~5 cm^3^ of sediment from the centre of one core section half into a sterile 15 mL centrifuge tube using a sterile plastic spatula. Duplicate samples were collected per depth interval (Table 1) and frozen at −80 °C.

**Table 1:** Santa Barbara sediment core sample details and associated age model estimates.

MV1012-002P section	Section age (at bottom of section) (ka)	Depth (cm) from top of each 25 cm subsample	*sed*aDNA extract ID (duplicates)	Stratigraphic depth (mbsf)	Age estimate (ka)
1 1.25–1.5 mbsf	~0.75–0.9	0	55, 56	1.25	0.761
3 4.25-4.5 mbsf	~2.7–2.9	10	41, 42	4.35	2.844
5 7.25-7.5 mbsf	~4.8–4.9	10	29, 30	7.35	4.860
8 11.75-12.0 mbsf	~7.8–8.0	10	19, 20	11.85	7.884
11 16.25-16.5 mbsf	~10.8–11.0	20	1, 2	16.45	10.975
EBC			57, 58		

The age-depth model was based on the previously developed age-depth model for site ODP Site 893 A, which is located <100 m away from our coring site MV1012-002P and has approximately identical sedimentation history [34]. *EBC* Extraction blank control; *ka* thousand years ago; *mbsf* metres below seafloor.

An independent age model does not exist for Core MV1012-002P, thus we applied an approximate age-depth model from Ocean Drilling Program (ODP) Site 893 A [34, 35], located less than 100 m from Core MV1012-002P (Table 1). Cores collected in this region have similar age-depth models [36], and correlation between cores is generally within the error range of Δ^14^C radiocarbon-based ages.

### *sed*aDNA extractions

Hoods and equipment were de-contaminated before and after extractions (using Surfa’Safe Premium and UV light). Gloves were frequently changed, and equipment and surfaces were disinfected between processing each sample. One extract was prepared for each sample (i.e., two extracts per depth, Table 1) from ~0.25 g of sediment, working from the oldest to youngest sample. Extractions followed the DNeasy PowerLyzer PowerSoil DNA Isolation Kit (QIAGEN, Germany) protocol, except that DNA was eluted three times in 60 µL elution buffer instead of once in 100 µL to achieve a higher DNA concentration. We added two extraction blank controls (EBCs; extracts 57,58) by treating empty bead-tubes with the same protocol, which provided a total of 12 extracts (10 samples, 2 EBCs). Library preparation and sequencing of the EBCs followed the same procedure as for samples.

### Metagenomic (‘shotgun’) library preparation

Libraries were prepared from the 12 raw extracts using the TruSeq Nano DNA Low Throughput Library Prep Kit (Illumina, CA, USA) with TruSeq DNA Single Indexes Set A (Illumina). We followed the manufacturer’s protocol, except that we retained all DNA fragments by not removing large fragments and by adding 200 µL Sample Purification Beads (instead of 30 µL as per Illumina protocol) in the “small fragments removal” step. Instead of purifying our libraries using magnetic beads we ran them on a 1.5% agarose gel and cut out bands between 200 - 300 bp using sterile scalpels. We pooled the gel pieces of our duplicate libraries in one vial and purified them using the NucleoSpin Gel and PCR Clean-up kit and protocol (Macherey Nagel, Germany). We washed and eluted the DNA twice with the same 12 µL elution buffer and quantified the libraries using the Qubit dsDNA HS Assay (Invitrogen, MA, USA). The DNA-content of library of sample 41/42 (4.35 mbsf) was very low, thus we added an ethanol precipitation step (final volume 6 µL), and then pooled the barcoded libraries into an equimolar 10 nM pool (except for sample 41/42, 4.35 mbsf, which was 7.64 nM). The samples were sequenced on a HiSeq 4000 (2 ×150 bp cycle; ~350 Mio paired-end reads total, i.e., ~58Mio/sample, and an approximate sequencing depth of 20X/sample assuming a diatom genome-size of 80–100 Mb) at Fasteris, Switzerland.

### Metabarcoding (‘amplicon’) library preparation

We amplified the *18S-V9* region (121 bp) using PCR (25 µL/reaction) containing 1 µL DNA template (1 in 10 dilution), Pfu Buffer (final concentration 1X, 2.5 µL) and Pfu Polymerase (1.25 units, 0.2 µL) (Promega, WI, USA), dNTPs (10 mM each, 0.5 µL), the primer pair 1389 F 5′-TTGTACACACCGCCC-3′ and 1510 R 5′-CCTTCYGCAGGTTCACCTAC-3′ (0.3 µM, 1 µL each) [15], and nuclease-free water (18.8 µL). PCR amplifications (lid-preheat to 105 °C, 30 s at 98 °C; 35 cycles of 10 s at 98 °C, 30 s at 57 °C, 30 s at 72 °C; and 72 °C for 10 min) were performed in triplicates on a Mastercycler (Eppendorff, Germany) and then pooled.

We amplified the *diat-rbcL* region (76 bp) using PCR (25 µL/reaction) containing 1 µL DNA template (1 in 10 dilution), PCR Buffer II (final concentration 1X, 2.5 µL) and MgCl_2_ (1.5 mM, 1.5 µL) and AmpliTaq Gold Polymerase (1.25 units, 0.125 µL) (Applied Biosystems, MA, USA), dNTPs (10 mM each, 0.5 µL), the primer pair Diat_rbcL_705F (AACAGGTGAAGTTAAAGGTTCATAYTT) and Diat_rbcL_808R (TGTAACCCATAACTAAATCGATCAT), (0.32 µM, 0.8 µL each) [29], and nuclease-free water (17.78 µL). PCR amplifications (lid-preheat to 105 °C, 8 min at 95 °C; 45 cycles of 10 s at 95 °C, 30 s at 43.6 °C, 30 s at 72 °C; and 72 °C for 10 min) were done in triplicates and pooled.

PCRs were set up in the paleogenomics lab, and then run in a physically separated post-PCR lab. Library preparation principally followed *the protocol described above for the shotgun libraries*, using 10 µL of each sample’s *18S-V9* PCR product mixed with 6.25 µL (to achieve an equimolar concentration) of each sample’s *diat-rbcL* PCR product diluted with nuclease-free water to a final library volume of 60 µL. DNA bands between 150 and 200 bp were cut from the gel, with the replicates per sample pooled, cleaned up and quantified as described in *2.3*. Sequencing was undertaken using a MiSeq Nano V2 2 ×125 bp cycle; ~1 Mio paired-end reads total, with ~166,000/sample shared sequencing run containing both *18S-V9* and *diat-rbcL* amplicons, providing a sequencing depth of 2 371X for *diat-rbcL* (assuming 134 diatom species, see Results) and 619X for *18S-V9* (assuming 35 phyla, see Results) at Fasteris.

### Bioinformatics and statistical analyses

We received already demultiplexed raw sequencing data (see Supplementary Material for sequencing output), which we processed using the same parameters for shotgun and amplicons, following the marine eukaryote *sed*aDNA bioinformatic pipeline described in [10] (and Supplementary Material). To investigate the eukaryote composition, we processed both shotgun and amplicon data by comparing to a PR^2^-derived V9 database, namely *V9_PR2* [16]. We subtracted species identified in EBCs (Supplementary Material Table 1) and exported read counts per sample on phylum-level (all nodes). To be able to visualise the data, we selected all eukaryote taxa that occurred with a relative abundance of >0.1% (which together made up >99% of the community) in each of the two datasets (31 and 27 taxa in the shotgun and amplicon data, respectively).

We tested whether a relationship exists between the average *V9_PR2* reference sequence length for the more abundant taxa and their over/-underrepresentation in amplicon relative to shotgun data (as per [26] for *16S-V3*). For this, we extracted reference sequence length distribution data (‘abundant’ taxa identified in both shotgun and amplicon) from the *V9_PR2* database (Supplementary Material) and visualised this in a heatmap with the read counts data per taxon using the R library ggplot2 [37]. We drew the ratio between amplicon and shotgun (A:SG) read counts per abundant taxon per sample. As a few taxa had no read counts in some of the shotgun samples (Acantharea, Annelida, Basidiomycota, Chlorophyta, Chytridiomycota, and Opisthokonta) these taxa were excluded from the ratio, leaving 17 taxa for this analysis. We performed Pearson correlation analyses between the average read lengths (“PR2V9AL”) and the A:SG ratio per taxon per sample (PAST v.4.02 [38]) to test for overamplification of short reads in amplicon data. In addition, we compared read length (length of the aligned query/sample sequence) and coverage (how many bases were covered between query and reference sequence) (both exported from MEGANCE6-18-10) of all sequences assigned to Eukaryota, and also for Bacillariophyta, per sample.

For a detailed investigation of diatoms, we compared both the shotgun and amplicon data to an in-house *diat-rbcL* database (including 1 472 unique 76 bp long *diat-rbcL* sequences, Supplementary Material). Alignments and EBC taxa filtering were done as for *V9_PR2* (Supplementary Material Table 2). Read length and coverage were extracted from.blastn-files and MEGAN, respectively. Correlation analysis on *diat-rbcL* reference sequence lengths and over-/underrepresentation of diatoms was not possible as for *V9_PR2* due to all *diat-rbcL* being 76 bp long. However, this data is provided with the Supplementary Material for completeness. Finally, we compared diatom composition as detected by *V9_PR2* and *diat_rbcL*, as well as the representation of the detected diatoms in each database to assess potential reference biases.

## Results

### Eukaryote composition (*V9_PR2*)

**Fig. 2:**
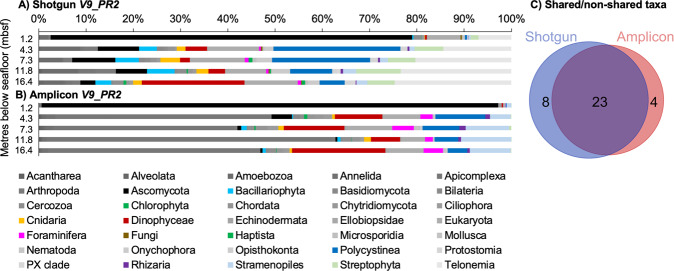
Eukaryote composition in five Santa Barbara Basin sediment samples post-alignment with *V9_PR2* database. Composition is shown in relative abundances for (**A**) shotgun, and (**B**) amplicon data (phylum-level). The surface sample should be considered with caution in both (**A**) and (**B**) due to the possibility of contamination (see “Methods”). **C** Venn diagram showing eukaryote taxa richness (phylum level) in the shotgun and amplicon data after alignment with the *V9_PR2* database (diagram areas are proportional to the total number of taxa included, for a list of shared/non-shared taxa see Supplementary Material Fig. 1). Only taxa abundant on average >0.1% are included, as they make up >99% of the eukaryote composition.

Using *V9_PR2* we were able to assign a total of 15 668 (shotgun) and 90 689 reads for the shotgun and amplicon data, respectively. These reads represented 14%, 54%, 0 and 32% (shotgun), and 0%, 29%, 0 and 71% (amplicon) unassigned cellular organisms, Bacteria, Archaea and Eukaryota, respectively. Within the eukaryotes, we determined 51 and 64 taxa for shotgun and amplicon data, respectively. Abundant taxa (average abundance >0.1% across all samples; 31 and 27 taxa in shotgun and amplicon, respectively) are shown in Fig. 2. The latter includes 23 taxa (including assignments made on “Eukaryota” level) that were shared between shotgun and amplicon, and four taxa only detected in the amplicon data (Fig. 2C).

Within shotgun, the most abundant eukaryotes were Ascomycota (53%), Telonemia (11%), Eukaryota (not further determined, 8%), Polycystinea (4%), Dinophyceae (3.8%), Streptophyta (3.2%), Amoebozoa (3%), Cercozoa (1.6%), Bacillariophyta (1.6%), Arthropoda (1%). In the amplicon data, the most abundant eukaryotes were Ascomycota (33%), Apicomplexa (30%), Dinophyceae (9.5%), Stramenopiles (6.3%), Eukaryota (4.9%), Polycystinea (3.5%), Foraminifera (3.2%), Cercozoa (1.1%) and Chordata (1%). Thus, a total of 10 and 9 taxa were abundant with >1% (average across all samples) in the shotgun and amplicon data, including only five taxa (Ascomycota, Eukaryota, Dinophyceae, Polycystinea, Cercozoa) that were picked up by both methods (i.e., are amongst the shared taxa in Fig. 2C, Supplementary Material Fig. 1). Taxa detected by one method or the other were slightly rarer species (between 0.1 and 1% average relative abundance across all samples; Supplementary Material Table 3).

The shotgun EBC detected two taxonomic groups, one prokaryotic (Gammaproteobacteria) and one eukaryotic (Poacea). The amplicon EBC detected 46 taxa, of which 12 were prokaryotes and 34 were eukaryotes, including dinoflagellate taxa (*Dinophysis* and *Alexandrium*), Calanoida and Bacillariophyta (copepods and diatoms, respectively; Supplementary Material Table 1). While any reads assigned to EBC taxa were removed from samples, including reads assigned to the Bacillariophyta node, reads assigned to Bacillariophyta at lower taxonomic levels (e.g., Bacillariophycidae, Bacillariaceae, etc.) remain summarised under the phylum-level Bacillariophyta node (Fig. 2).

### Relationship between Eukaryota composition and *V9_PR2* reference sequence length

**Fig. 3:**
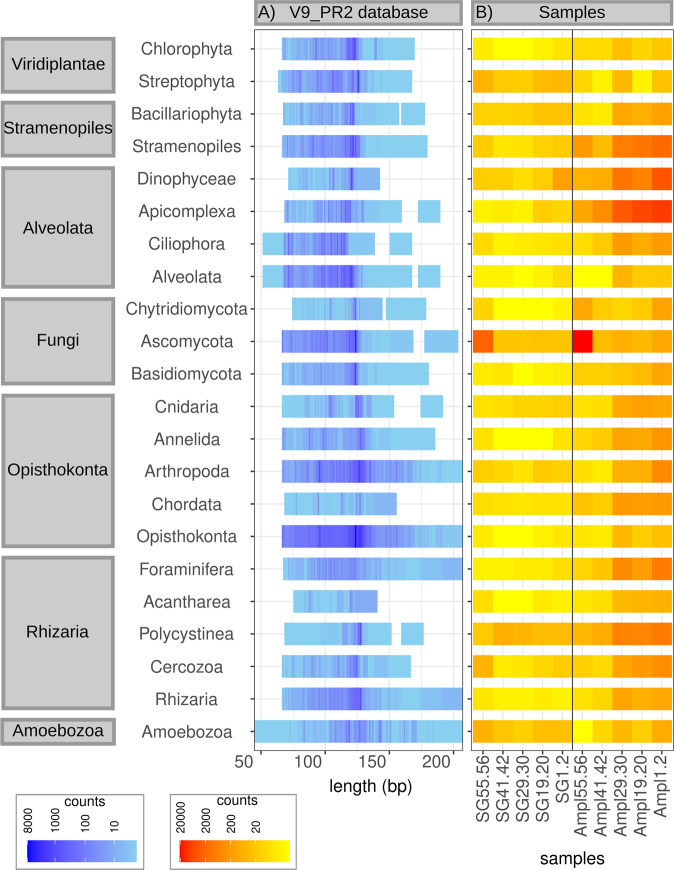
Average sequence lengths for individual eukaryote taxa as per in the *V9_PR2* database (A) and read counts for these taxa in shotgun (SG) and amplicon (Ampl) data (B). Listed are all taxa that occurred on average >0.1% across all samples in either the shotgun or amplicon dataset, or both. Only taxa that were determined in both shotgun and amplicon data are included.

*V9_PR2* reference sequence-lengths for the relatively abundant taxa (>0.1% across all samples, including all taxa that were shared and assigned below eukaryote-level, i.e., 22 taxa, see Supplementary Material Table 3) were around the overall average sequence length of the *V9_PR2* database (121 bp) (Fig. 3). However, considerable length variation was observed, with most of the abundant taxa being represented by shorter than average reference sequences in the *V9_PR2* database, and a few taxa (e.g., Arthropoda, Opisthokonta and Amoebozoa) with a number of reference sequences longer than average (Fig. 3).

We determined a negative correlation between the average *V9_PR2* reference sequence length (V9PR2AL) and the A:SG read counts ratio per taxon for all samples (*r*_V9PR2AL,A:SG_1.2_ = −0.27269, *r*_V9PR2AL,A:SG_4.3_ = −0.33233, *r*_V9PR2AL,A:SG_7.3_ = −0.28064, *r*_V9PR2AL,A:SG_11.8_ = −0.32559, *r*_V9PR2AL,A:SG_16.4_ = −0.30078). This means that shorter *V9_PR2* reference sequences for our abundant taxa were associated with an overamplification of these taxa in the amplicon data (for average *V9_PR2* reference sequence length of the abundant taxa and A:SG ratios see Supplementary Material Table 4).

### Eukaryota and Bacillariophyta sequence length and coverage post-*V9_PR2* alignment

Sequences assigned to Eukaryota in shotgun were on average 112 bp and in amplicon data 161 bp, i.e., shotgun reads were around ~50 bp shorter than amplicon reads (Table 2). Bases covered in shotgun were ~40 bp shorter than in amplicon data (Table 2). Similarly, sequences assigned to Bacillariophyta were on average 124 and 167 bp in shotgun and amplicon data, respectively, so showed an ~40 bp difference. For Eukaryota, there was a difference of ~23 bp and 29 bp between sequence length and coverage in shotgun and amplicon data, respectively. For Bacillariophyta, we found a ~36 and ~37 bp difference between sequence length and coverage in shotgun and amplicon data, respectively.

**Table 2:** Lengths and coverage of sequences assigned to Eukaryota and Bacillariophyta in shotgun and amplicon data.

	Shotgun (read length)		Amplicon (read length)		Shotgun (bases covered)		Amplicon (bases covered)	
	Average	StDev	Average	StDev	Average	StDev	Average	StDev
*Eukaryota*								
All samples	112	28	161	13	89	28	132	19
1.25 mbsf	110	26	169	4	99	27	149	4
4.35 mbsf	118	27	161	14	79	33	127	18
7.35 mbsf	109	28	161	13	86	34	126	16
11.85 mbsf	122	32	156	14	85	38	120	17
16.45 mbsf	118	28	160	13	92	35	124	15
*Bacillariophyta*								
All samples	124	27	167	2	88	23	130	1
1.25 mbsf	119	19	168	0	86	15	130	0
4.35 mbsf	143	31	167	2	85	27	129	2
7.35 mbsf	112	13	167	2	98	17	130	1
11.85 mbsf	116	25	167	3	94	27	130	1
16.45 mbsf	123	21	167	2	99	23	130	1

Listed are lengths (Average and Standard Deviation, StDev) and coverage (bases covered) of sequences assigned to Eukaryota (top) and Bacillariophyta (bottom) after alignment to the *V9_PR2* database.

Bacillariophyta read lengths and coverage were similar to those of Eukaryota, for both shotgun and amplicon data (Table 2). Variation in sequence lengths and coverage was much higher in shotgun than in amplicon data. We found no trend towards shorter (i.e., more fragmented) sequences with increasing subseafloor depth for either Eukaryota or Bacillariophyta in the shotgun data. Eukaryota shotgun read lengths were on average ~9 bp shorter (112 bp) than the average reference sequences in the *V9_PR2* database (121 bp).

### Diatom composition detected via *diat-rbcL* and read length characteristics

**Fig. 4:**
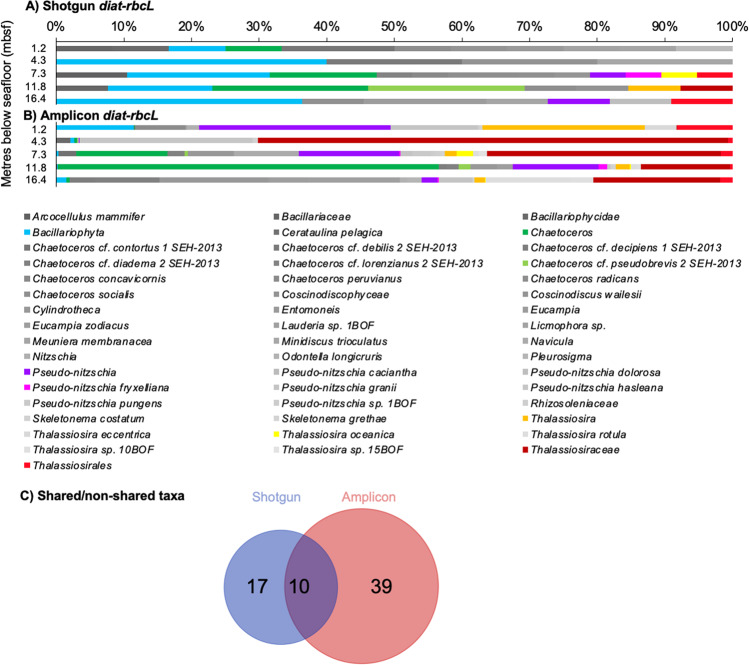
Diatom composition in the Santa Barbara Basin sediment samples post-alignment with *diat-rbcL* database. Diatom composition is shown as relative abundance for (**A**) shotgun and (**B**) amplicon data. The surface sample should be considered with caution in both (**A**) and (**B**) due to the possibility of contamination (see “Methods”). **C** Venn diagram showing diatom taxa richness (species level) in the shotgun and amplicon data after alignment with the *diat-rbcL* database (diagram areas are proportional to the total number of taxa included, for a list of shared/non-shared taxa see Supplementary Material Fig. 2). Only taxa abundant on average >0.1% are included (in **A**, **B**, **C**).

A total of 60 (shotgun) and 80 674 (amplicon) reads were assigned to diatoms (Fig. 4). In total, 27 taxa were determined in the shotgun, and 140 in the amplicon dataset. When considering the “abundant” taxa (on average >0.1%), 27 and 49 diatoms were determined in the shotgun and amplicon data, respectively (Fig. 4). A total of 10 taxa were shared between the two datasets *Bacillariophyta, Bacillariophycidae, Chaetoceros, C*. cf. *pseudobrevis 2 SEH-2013, Pseudo-nitzschia, P. fryxelliana, Thalassiosiraceae, Thalassiosirales, Thalassiosira* and *T. oceanica* (Fig. 4C, Supplementary Material Fig. 2). Sequences assigned to diatoms via *diat-rbcL* were shorter (by ~16 bp) in the shotgun than in the amplicon data, with amplicon read lengths and coverage all 76 + 1 bases (Table 3).

**Table 3:** Bacillariophyta sequence lengths in shotgun and amplicon datasets.

	Shotgun (read length)		Amplicon (read length)		Shotgun (covered bases)		Amplicon (covered bases)	
	Average	StDev	Average	StDev	Average	StDev	Average	StDev
Bacillariophyta (all samples)	60	15	76	1	59	16	76	1
1.25 mbsf	64	14	76	1	64	13	76	1
4.35 mbsf	58	9	76	1	54	4	76	1
7.35 mbsf	59	15	76	1	62	16	76	1
11.85 mbsf	59	17	76	1	52	17	76	1
16.45 mbsf	60	16	76	1	56	16	76	1

Averages and standard deviations (StDev) for all reads assigned to Bacillariophyta (read lengths and bases covered), overall (all samples) and for each individual sample (mbsf = metres below seafloor).

No diatoms were detected in the shotgun EBC, however, 45 taxa were determined in the amplicon EBC with most reads assigned to *Chaetoceros* spp. (especially, *Chaetoceros debilis*, *C. socialis* and *C. radicans*), several *Thalassiosira* and *Pseudo-nitzschia* species, as well as others (Supplementary Material Table 2).

### Comparison of *V9_PR2* vs. *diat-rbcL* derived diatom composition

**Fig. 5:**
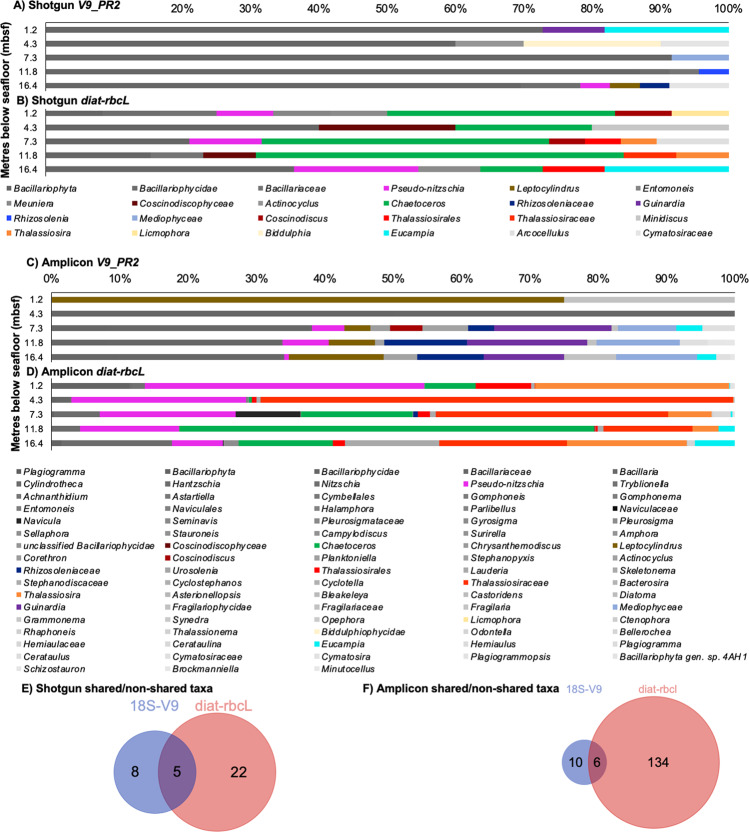
Comparison of diatom composition in Santa Barbara Basin sediment samples determined in shotgun and amplicon data using the *V9_PR2* and *diat-rbcL* databases. Relative abundance of diatoms (genus level) in the shotgun data after aligning to (**A**) *V9_PR2* and (**B**) *diat-rbcL*. Relative abundance of diatoms (genus level) in the amplicon data after aligning to (**C**) *V9_PR2* and (**D**) *diat-rbcL*. The surface sample should be considered with caution in (**A**–**D**) due to the possibility of contamination (see “Methods”). Venn diagrams of shared and non-shared diatom taxa after alignment to the *V9_PR2* (18S-V9) and *diat-rbcL* databases for the shotgun (**E**) and amplicon (**F**) data (species level, diagram areas are proportional to the total number of species included). For a complete species list and their read counts per sample see Supplementary Material Fig. 3, Supplementary Material Table 5.

In the shotgun data, 79 and 60 sequences were assigned to diatoms using *V9_PR2* and *diat-rbcL* as the reference database, respectively, and composition differed considerably (Fig. 5). Using *V9_PR2*, diatoms were mostly assigned on relatively high taxonomic levels (e.g., Bacillariophyta) with few taxa being differentiated sporadically in the different samples (Fig. 5A, Supplementary Material Fig. 3). Using *diat-rbcL*, *Chaetoceros, Thalassiosira* and *Pseudo-nitzschia* were more prominent (Fig. 5B).

In the amplicon data, 329 sequences were assigned to diatoms using *V9_PR2*, and 80 674 using *diat-rbcL*. Using *V9_PR2*, few taxa were detected in the two top samples (*Leptocylindrus* and Fragilariaceae at 1.2 mbsf, Bacillariophycidae and Bacillariaceae at 4.3 mbsf) while the lowermost samples were more diverse (Fig. 5C). Using *diat-rbcL*, most reads were assigned to *Thalassiosira*, *Chaetoceros*, and *Pseudo-nitzschia*, with other taxa sporadically occurring at different depths (Fig. 5D). For a complete species list and their read counts see Supplementary Material Fig. 3, and Supplementary Material Table 5.

We found large differences in the number of shared vs. non-shared taxa between shotgun and amplicon data, and *V9_PR2* and *diat-rbcL* alignments (Fig. 5E, F). Database inspections showed that all taxa detected via *V9_PR2* were also represented in the *diat-rbcL* database, except Rhizosoleniaceae. However, out of the 22 taxa exclusively detected via *diat-rbcL* in shotgun (Fig. 5E, F), 10 are only represented in the *diat-rbcL* database (*Pseudo-nitzschia caciantha*, *P. dolorosa*, *Chaetoceros* cf. c*ontortus* 1 SEH-2013, *C*. cf. *lorenzianus* 2 SEH-2013, *C*. cf. *pseudobrevis* 2 SEH-2013, Thalassiosirales, Thalassiosiraceae, *Coscinodiscus wailesii, Arcocellulus mammifer, Meuniera membranacea*, Supplementary Material Fig. 3). Similarly, out of the 134 taxa exclusively detected via *diat-rbcl* in amplicon, 84 were in this database only, noticeably including several species and strains of *Chaetoceros, Pseudo-nitzschia, Thalassiosira* and *Cylindrotheca* (*eg*., additions SHE-2013, BOF in species names), amongst others (see Supplementary Material Fig. 3, Supplementary Material Table 5).

## Discussion

While previous studies have compared marine *sed*aDNA with microfossil records (e.g., [1, 39]), this is, to our knowledge, the first in-depth comparative analysis of shotgun and amplicon data derived from marine *sed*aDNA. We selected two very short gene regions (*18S-V9* ~121–130 bp, *diat-rbcL* ~76 bp) for our amplicon approach, anticipating that they would capture a similar breadth of eukaryote and diatom diversity as our shotgun data. However, taxonomic profiles differed considerably between the two approaches and with choice of alignment database (*V9_PR2, diat-rbcL*).

### Technical notes

Recently, protocols have been optimised for the extraction of marine eukaryote *sed*aDNA, achieving high yields of *sed*aDNA while also preserving the very small fragments typical of ancient DNA [10]. Our DNA extractions preceded these optimisations, using a protocol that may have produced a bias toward the longer spectrum expected for *sed*aDNA (~112 bp for eukaryotes in the shotgun data). Specifically, our protocol included DNA-binding spin columns, which have been shown to favour larger DNA fragments [40]. However, as we used the same protocol for all extractions, the comparisons between shotgun and amplicon data remain robust.

We determined a relatively high proportion of Fungi in sample 1.25 mbsf in both shotgun and amplicon data. Fungal growth can result from sub-optimal sediment core storage conditions, such as oxygen exposure [41], and it is likely that, in this sample, fungi had grown pre-extraction due to the damage of the core-section wrapping and oxygen exposure. Fungi presence in the other four samples was relatively low, indicating the extensive precautions to preserve the sediments anoxically over 7 years (bagging, flushing with nitrogen gas, adding oxygen absorbers, sealing and refrigeration) were adequate. While growth of anoxic bacteria during storage cannot be excluded [42], we would expect such growth to occur at very slow rates (as in sub-seafloor environments) with a minor impact on the here analysed eukaryote composition. Good preservation may have further contributed to our finding of relatively long sequences (~112 bp in shotgun) relative to other marine *sed*aDNA studies [10, 28].

### Eukaryote composition in shotgun and amplicon data

Eukaryote composition differed considerably between shotgun and amplicon data. We analysed relative compositional patterns at the phylum-level, with most (23) taxa detected by both datasets. However, the relative abundance of these shared taxa varied greatly. Based on *sed*aDNA fragment length, heatmap and correlation analyses, we showed that this difference was associated with the read lengths that are favoured by either of the two approaches (shotgun—variable, amplicon—prescribed).

Previously [26], showed that targeted amplification of the prokaryotic *16S-V3* gene region in ancient microbiome samples led to confounded taxonomic profiles. This result is due to the doubling-up of two systematic amplification biases; firstly, as gene regions are targeted that are longer than most sequences in the DNA extracts (assessable via shotgun data), and secondly, as shorter sequences (occurring at high abundance in ancient DNA samples) overamplify while longer sequences under-amplify relative to shotgun data. We found a negative correlation between average *V9_PR2* reference sequence length of our abundant eukaryotes and the A:SG read counts ratio for all samples. While these negative correlations were not significant, they were consistent with the results reported by [26] for *16S-V3*, and suggest a systematic amplification bias in our *18S-V9* amplicon data. It is possible that the ‘non-significance’ in our analyses was associated with *V9_PR2* reference sequences being much shorter (89–135 bp, ~45 bp range, for the abundant taxa) than *16S-V3* used by [26] (~145–215 bp, ~70 bp), providing a smaller bp range to influence correlation strength.

Read lengths were much shorter in the shotgun than in the amplicon data. This was expected, and most ancient sequences have been shown to be <100 bp [21, 22]. Amplicons define a specific DNA fragment size to be amplified, here being ~121–130 bp and 76 bp for *18S-V9* and *diat-rbcL*, respectively. One would assume that the closer an amplicon target gene region length is to the average DNA fragment lengths in a shotgun sample, the more similar the taxonomic profiles generated from shotgun and amplicon would be. This hypothesis was neither confirmed nor rejected by our data. Our raw filtered shotgun data (pre-alignment) provided an average sequence length of ~116 bp, which matched neither the length of *18S-V9* (~121–130 bp) nor that for *diat-rbcL* (76 bp). Amplicons achieved better coverage than shotgun data, which would generally be a clear advantage. However, if the compositional data is skewed due to amplification biases then this improvement is redundant. In the future, we recommend pursuing a metagenomic shotgun approach with high sequencing depth to avoid the biases, possibly coupled with hybridisation capture [43]. If a metabarcoding approach is used, exploratory shotgun analyses should precede this to determine average DNA fragment size and to guide target gene region length choices. However, the latter would not allow authentication of the ancient data in amplicons, as the DNA damage patterns underlying bioinformatic authentication assessments are read over during the amplification process [20].

### Diatom composition in shotgun and amplicon data

We expected some differences in species resolution between *18S-V9* and *diat-rbcL*, and possibly higher resolution in *diat-rbcL* due to *rbcL*’s demonstrated capability to distinguish phytoplankton at species level [30, 31]. Comparing diatom composition post *V9_PR2* and *diat-rbcL* alignments revealed that about 1/2 to 1/3^rd^ of taxa was only represented in the *diat-rbcL* database, explaining the much higher species resolution via *diat-rbcL* in both shotgun and amplicon data (rather than species resolution due to the different markers per se). It also explained the overrepresentation of *Chaetoceros*, *Pseudo-nitzschia*, and *Thalassiosira* in *diat-rbcL* relative to *V9_PR2* data, exacerbated in amplicon data (134 diatoms were only detected via *diat-rbcL* in amplicon, compared to 22 taxa in the shotgun data - noting that the shotgun diatom results are based on very few assigned reads (<80 each)). While a detailed comparison of our genetic data with existing Santa Barbara Basin diatom microfossil records exceeds the scope of this study, the shotgun data appeared to have broadly captured relative abundances of diatoms expected as per such records [8, 9, 44].

All diatoms detected via *V9_PR2* (except Rhizosoleniaceae) were also represented in the *diat-rbcL* database. Yet, some of these diatoms (8 taxa in shotgun, 10 in amplicon) were not detected via *diat-rbcL*. Potential reasons why these diatoms were not detected by *diat-rbcL* might include the overrepresentation of nuclear (here, *18S-V9*) relative to chloroplast DNA (here, *diat-rbcL*), for example, due to faster degradation of chloroplast DNA, as has been shown for the phytoplankton *Euglena gracilis* [45]. It is also possible that chloroplast DNA was low in our samples, at least for some species, as its amount depends on species-specific chloroplast-size [46]. However, very little is known about marine eukaryote *sed*aDNA degradation (chloroplast and nuclear) with time, sediment properties, species specificity, and this requires further research. In any case, the continued improvement of reference databases through sequence additions is crucial to generate comprehensive *sed*aDNA taxonomic profiles.

### Extraction blank controls

We detected few contaminant taxa in the shotgun data, whereas the high number of eukaryotes and diatoms determined in the amplicon EBCs (34 and 45 taxa, respectively) was concerning. Amongst these amplicon contaminants were common modern ocean protist species often used as environmental indicators (e.g.*, Alexandrium*—eutrophication, *Dinophysis*—tropicalisation [47], *Chaetoceros*—open ocean and upwelling conditions [48]). Diatoms identified in our *diat-rbcL* EBCs, including various *Chaetoceros* and *Thalassiosira* species, were also detected in controls by [33], who used the same *diat-rbcL* marker to investigate Fram Strait paleo-diatoms over 30,000 years. These matches even included the exact same sequences for some species (e.g.*, Chaetoceros* cf. *contortu*s 1 SEH-2013, *Actinocyclus* sp. 1 MPA-2013). This demonstrates the importance of EBC inclusion to track common contaminants and assess which species might have been identified based on PCR artefacts. We acknowledge that processing EBC’s will incur additional costs. However, it will significantly improve the interpretation of results.

## Conclusion

Our comparison of paleo-eukaryote, especially diatom, composition via metabarcoding and shotgun metagenomics showed considerable differences in taxonomic profiles (including EBC profiles), which were related to differences in sequence length distributions, and influenced by the choice of reference database (*18S-V9*, *diat-rbcL)*. We conclude that deep metagenomic sequencing remains the most suitable and unbiased approach to study marine eukaryote *sed*aDNA. If metabarcoding is the chosen technique for a given study, then this should be combined with shotgun metagenomics, at least of a few samples, to determine the bias expected from the difference in target gene region length and average length as per shotgun metagenomics.

## Supplementary information

Supplementary Material

## Data Availability

The raw data (shotgun and amplicon) are publicly available via the NCBI Sequence Read Archive (SRA) under BioProject accession number PRJNA766251 (“Santa Barbara Basin *sed*aDNA”, Sep 21).
