# Supplementary material for: Paleo-diatom composition from Santa Barbara Basin deep-sea sediments: a comparison of 18S-V9 and diat-rbcL metabarcoding vs shotgun metagenomics
Source: ISME Commun. 2021 Nov 9;1:66. doi: 10.1038/s43705-021-00070-8 (PMC9723766; doi:10.1038/s43705-021-00070-8)
Supplement: Supplementary file 1 — Supplementary Material [file 43705_2021_70_MOESM1_ESM.pdf]

Supplementary Material

Paleo-diatom composition from Santa Barbara Basin deep-sea sediments: A comparison of *18S-V9* and *diat-rbcL* metabarcoding vs shotgun metagenomics

Authors: Linda Armbrecht, Raphael Eisenhofer, José Utge, Elizabeth C. Sibert, Fabio Rocha, Ryan Ward, Juan José Pierella Karlusich, Leila Tirichine, Richard Norris, Mindi Summers, Chris Bowler

Table of contents:

Content	Page
Bioinformatic processing of shotgun and amplicon data	1
Relationship between *diat-rbcL* reference sequence length and diatom composition	2
Supplementary Material Table 1: Taxa detected in extraction blank controls (EBCs) post *V9_PR2* alignment.	3
Supplementary Material Table 2: Diatoms detected in extraction blank controls (EBCs) post *diat-rbcL* alignment	4
Supplementary Material Fig. 1: Eukaryota relative abundance in shotgun and amplicon data after alignment with *V9_PR2* database	5
Supplementary Material Table 3: Total and relative abundance of eukaryote taxa identified in the shotgun and amplicon data post *V9_PR2* alignment	5
Supplementary Material Table 4: Correlation analyses (parameters and statistics)	6
Supplementary Material Figure 2: Diatom relative abundance in shotgun and amplicon data after alignment with *diat-rbcL* database	7
Supplementary Material Figure 3: Comparison of diatom relative abundance in shotgun and amplicon data after alignment with *V9_PR2* and *diat-rbcL* database	8
Supplementary Material Table 5. Diatom read counts in shotgun and amplicon data after alignment with *V9_PR2* and *diat-rbcL* database	9
References	12

Bioinformatic processing of shotgun and amplicon data

Fasteris provided us with already demultiplexed raw sequencing data. For the shotgun samples, the amount of sequence obtained for each sample were: Sample 1,2 =26'284 Mb, Sample 19,20 = 20'171 Mb, Samples 29,30 = 19'025 Mb, Sample 41,42 = 11'987 Mb, Sample 55,56 = 28'860 Mb, Sample 57,58 = 39 Mb. For the amplicons (combined amount of *diat-rbcL* and *18S-V9*) sequence obtained for each sample were: 72 Mb, 41 Mb, 48 Mb, 18 Mb, 12 Mb, 32 Mb (same sample order). This corresponded to ~87.6, 67.2, 63.4, 40, 96.2, 0.1 Mio single-end reads (all on average 150 bp long) for shotgun, and to ~0.3, ~0.2, ~0.2, ~0.1, ~0.1, ~0.2 Mio SE reads (125 long) for amplicon samples, respectively.

We processed this raw sequencing data using the same parameters for shotgun and amplicon, following the marine eukaryote *seda*DNA bioinformatic pipeline described in Armbrecht et al. (2020). In brief, we trimmed adapters and merged reads using AdapterRemoval 2.30 (allowing a barcode mismatch of 1 bp and discarding reads <25 bp; Schubert et al., 2016), removed low-

complexity sequences (Komplexity software, threshold 0.55; Clarke et al., 2019) and, for the shotgun data, removed duplicated sequences using the dedupe tool in BBMap 38.58. FastQC (version 0.11.8; Babraham Bioinformatics) and MultiQC (version 1.0.dev0; Ewels et al., 2016) were run on all merged reads before and after filtering.

To investigate the eukaryote composition, we processed both shotgun and amplicon data by comparing to a PR^2-derived V9 database, namely *V9_PR2* (De Vargas et al., 2015). Using MALT, we built an index for this database and performed semiglobal alignments (Herbig et al., 2016). We converted the aligned sequence files (.blastn) to .rma6 format using the Blast2RMA tool in MEGAN6 (version 6_15_1) (Huson et al., 2016) with the default settings except for a minimum support percent of zero ('off'), a minimum bit score of 50, an E-value of 0.01, and a minimum percent identity of 95%. For our heatmap, we extracted reference sequence lengths for each 'abundant' taxon (identified in both shotgun and amplicon) from the *V9_PR2* database using BBMap's (https://sourceforge.net/projects/bbmap/) filterbyname.sh script. Sequences >200 bp were discarded using the reformat.sh script, before sequence lengths were calculated using the readlength.sh script.

For both the amplicon and shotgun data we performed subtractive filtering (i.e., subtracting reads for species identified in EBCs from samples) in MEGANCE6-18-10. All taxa identified in the EBCs (2 taxa in the shotgun, 46 taxa in the amplicon data) are listed in Supplementary Material Table 1; and hereafter, the term 'samples' refers to sediment-derived data post-EBC subtraction. Next, we exported read counts per sample on phylum-level (all nodes). We grouped the two categories 'Stramenopiles' and 'unclassified Stramenopiles' into one.

For a more detailed investigation of diatoms, we also compared both our shotgun and amplicon data to an in-house *diat-rbcL* database. We built this database by downloading all the diatom *RbcL* protein sequences from Uniprot database (https://www.uniprot.org, last accessed November 2020) and used them as queries for retrieving the corresponding nucleotide sequences from NCBI (http://www.ncbi.nlm.nih.gov, last accessed November 2020) from which we extracted the 76 bp region that we had targeted with our *diat-rbcL* primers and we then reduced redundancy with CD-HIT version 4.6.4 using a 100% identity cut-off (Li and Godzik, 2006). We built a MALT-index for this database and aligned our sequences as described for the *V9_PR2* database. Species detected in the EBC (none for shotgun) were subtracted from the samples (MEGANCE6-20-19); and listed in Supplementary Material Table 2.

Relationship between *diat-rbcL* reference sequence length and diatom composition

Testing the relationship between *diat-rbcL* reference sequence length and over/-underrepresentation of diatom species in amplicon relative to shotgun data was not possible as for eukaryotes (above). Firstly, we were unable to generate an A:SG read counts ratio per taxon due to the large differences in species determined by shotgun and amplicon data, and due to absence of species-level assignments in the shotgun data, which were primarily determined on higher taxonomic levels (thus, the A:SG ratio was indeterminable for all taxa except 'Bacillariophyta' for which $A:_{SG1.2mbsf}$=607, $A:SG_{4.3mbsf}$=10, $A:_{SG7.3mbsf}$=12.75. $A:SG_{11.8mbsf}$=8, $A:SG_{16.4mbsf}$=116.75). Secondly, all reference sequences in our *diat-rbcL* database were 76 bp in length, which prevented correlations.

Supplementary Material Table 1: Taxa detected in extraction blank controls (EBCs) post *V9_PR2* alignment. The number of reads detected is also provided. All taxa listed were removed from the downstream analyses of the *V9_PR2* samples (separately for Shotgun and Amplicon data).

**Shotgun EBC taxa**	**Read counts**	**Amplicon EBC Taxa**	**Read counts**
Prokaryota		Prokaryota	
Gammaproteobacteria	1	*Hyphomicrobium*	1
		Belnapia	1
		Sphingomonadaceae	213
		Desulfovibrio	2
		Enterobacterales	1
		Oceanospirillales	1
		Acinetobacter	199
		Alkanindiges	1
		Pseudomonas	147
		Francisella	1
		Opitutus	193
		Clostridium	7
Eukaryota		Eukaryota	
Poaceae	1	*Cephaloidophora*	26
		Mesodiniidae	1
		Salpingella	1
		Strombidium	1
		Dinophysis	1
		Alexandrium	1
		Hematodinium	1
		Protophysarum	1
		Discoba	7
		Pleosporales	1
		Penicillium	47
		Pseudogymnoascus	5
		Thelebolus	1
		Metschnikowia	5
		Cryptococcus	1
		Ustilaginaceae	5
		Chytridiomycota	2
		Oikopleura	1
		Platyhelminthes	1
		Calanoida	1
		Tellinoidea	1
		Siphonophorae	4
		Ichthyosporea	2
		Chaunacanthida	3
		Pyrgo	2
		Quinqueloculina	2
		Bolivina	1
		Globigerinita	1
		Neogloboquadrina	1
		Collodaria	4
		Actinommidae	4
		Larcopyle	2
		Bacillariophyta	1
		Pentapetalae	1

Supplementary Material Table 2: Diatoms detected in extraction blank controls (EBCs) post *diat-rbcL* alignment. The number of reads detected per taxon is also provided. All taxa listed were removed from the downstream analyses of the *diat-rbcL* amplicon data. No diatoms were detected in the EBC of the shotgun data after alignment to the *diat-rbcL* database.

Diatom taxon determined in diat-rbcL Amplicon EBC	Number of reads
Cylindrotheca closterium lineage V	1
Cylindrotheca sp. 2BOF	1
Cylindrotheca sp. lineage II	2
Cylindrotheca sp. lineage IV	1
Nitzschia cf. *laevis*	7
Pseudo-nitzschia caciantha	6
Pseudo-nitzschia delicatissima	3
Pseudo-nitzschia dolorosa	10
Pseudo-nitzschia galaxiae	14
Meuniera membranacea	2
Navicula cf. *perminuta*	6
Navicula tripunctata	1
Gyrosigma sp. 1BOF	1
Attheya septentrionalis	1
Chaetoceros cf. *contortus* 1 SEH-2013	7
Chaetoceros cf. *debilis* 2 SEH-2013	62
Chaetoceros cf. *decipiens* 2 SEH-2013	2
Chaetoceros cf. *diadema* 1 SEH-2013	3
Chaetoceros cf. *lorenzianus* 2 SEH-2013	6
Chaetoceros cf. *pseudobrevis* 1 SEH-2013	3
Chaetoceros didymus	8
Chaetoceros radicans	26
Chaetoceros similis	5
Chaetoceros simplex	2
Chaetoceros socialis	56
Corethron sp. 1BOF	2
Actinocyclus sp. 1 MPA-2013	5
Melosira sp. 2BOF	17
Pseudosolenia calcar-avis	1
Rhizosolenia setigera	6
Lauderia	7
Skeletonema	5
Detonula pumila	2
Minidiscus spinulatus	4
Minidiscus trioculatus	2
Thalassiosira aestivalis	1
Thalassiosira angulata	1
Thalassiosira anguste-lineata	22
Thalassiosira minima	1
Thalassiosira minuscula	1
Thalassiosira sp. 11BOF	1
Thalassiosira sp. 12BOF	1
Asterionellopsis glacialis	1
Eucampia	13
Arcocellulus mammifer	2

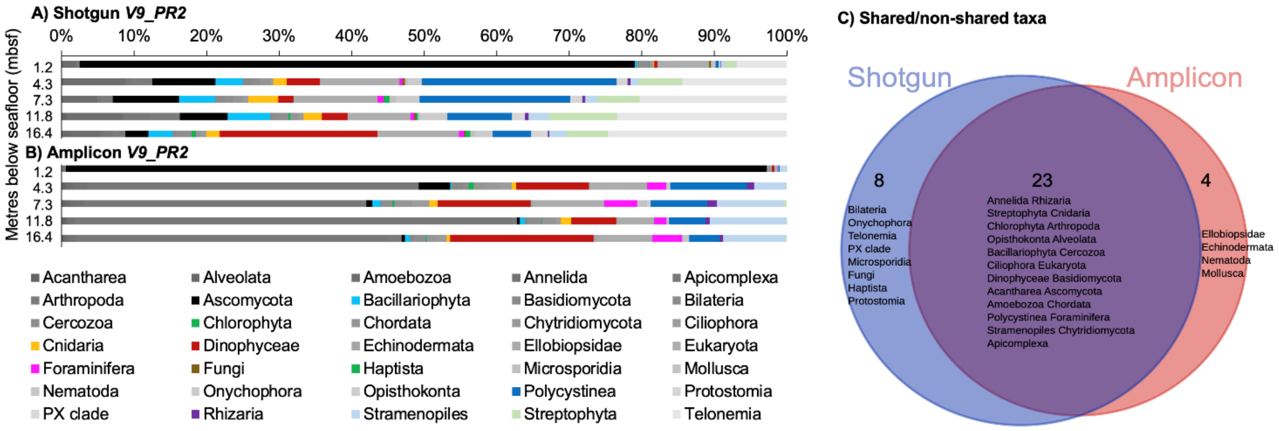

Supplementary Material Fig. 1. Eukaryota relative abundance in shotgun and amplicon data after alignment with *V9_PR2* database. Taxa are listed on phylum level, with A) and B) corresponding to Main Text Fig. 2, and C) listing the individual taxa names. See Supplementary Material Table 3 for list of taxa names and read counts.

Supplementary Material Table 3: Total and relative abundance of eukaryote taxa identified in the shotgun and amplicon data post *V9_PR2* alignment. Taxa are listed on phylum level, with read counts per sample provided as well as average relative abundance across all samples.

	Shotgun readcounts					Rel. abundance (%)	Amplicon readcounts					Rel. abundance (%)
SampleID	SG55-56	SG41-42	SG29-30	SG19-20	SG1-2	SG average proportion across all samples	Ampl55-56	Ampl41-42	Ampl29-30	Ampl19-20	Ampl1-2	Amplicon average proportion across all samples
Depth (mbsf)	1.2	4.3	7.3	11.8	16.4		1.2	4.3	7.3	11.8	16.4	
Acantharea*	5	0	0	2	1	0.16	3	5	42	57	68	0.27
Alveolata*	1	1	0	1	6	0.18	0	0	54	16	18	0.14
Amoebozoa	61	22	12	30	29	3.08	0	8	41	13	83	0.23
Annelida*	4	0	0	0	4	0.16	9	17	135	104	250	0.80
Apicomplexa	1	2	1	14	9	0.54	116	390	3427	6553	9042	30.40
Arthropoda	13	8	4	17	12	1.08	5	2	66	70	460	0.94
Ascomycota	2580	23	22	26	22	53.43	21122	37	69	43	103	33.27
Bacillariophyta	11	10	12	23	23	1.58	4	2	105	74	144	0.51
Basidiomycota*	2	3	0	1	2	0.16	13	13	22	37	108	0.30
Bilateria^*	0	1	3	1	1	0.12						
Cercozoa	52	2	3	8	15	1.60	12	7	122	214	362	1.12
Chlorophyta*	2	0	0	1	4	0.14	7	6	30	9	20	0.11
Chordata	5	4	3	4	4	0.40	25	14	214	181	223	1.02
Chytridiomycota*	10	0	0	0	1	0.22	98	15	8	16	134	0.42
Ciliophora	6	1	2	3	5	0.34	5	16	205	86	214	0.82
Cnidaria	4	5	10	10	13	0.84	4	5	104	154	113	0.59
Dinophyceae	14	12	5	14	150	3.90	61	86	1150	671	4190	9.59
Echinodermata^*							1	0	23	16	33	0.11
Ellobiopsidae^*							1	1	31	20	77	0.20
Eukaryota	239	29	28	34	78	8.16	104	68	852	538	1595	4.91
Foraminifera	1	1	2	2	5	0.22	9	22	408	176	877	2.32
Fungi^*	9	1	0	1	1	0.24						
Haptista^*	0	0	2	1	5	0.16						
Microsporidia^*	12	1	2	1	4	0.40						
Mollusca^*							6	3	119	25	94	0.38
Nematoda^*							0	0	0	0	84	0.13
Onychophora	7	5	8	14	14	0.96						
Opisthokonta*	2	0	0	1	3	0.12	3	2	47	13	30	0.15
Polycystinea	16	71	50	35	37	4.18	40	91	697	546	880	3.51
Protostomia^*	8	4	4	4	9	0.58						
PX clade^*	0	0	0	3	7	0.20						
Rhizaria	3	1	1	2	1	0.16	4	8	121	66	104	0.47
Stramenopiles	15	3	4	11	16	0.98	211	38	822	1154	1839	6.33
Streptophyta	57	16	14	37	40	3.28	8	1	43	1	28	0.13
Telonemia^*	234	38	49	92	171	11.67						

Circumflex (^) denotes taxa that were excluded from heatmap (non-shared and assignment made on 'Eukaryota' level).
Asterisk (*) denotes taxa excluded from correlation analyses (non-shared, assignment made on 'Eukaryota' level, and taxa for which the A:SG ratio couldnot be detrmined for each sample due to their abscence in some samples).

Supplementary Material Table 4: Correlation analyses. Input parameters for correlation analyses, the *V9_PR2* average reference sequence length (PR2V9AL) and amplicon:shotgun (A:SG) read counts ratio per taxon. Taxa for which no read count ratio was determinable (absence in some samples, see Suppl Table 2) were excluded. Pearson correlation coefficients (*r*) are also provided (Hammer et al., 2016; PASTv4.02).

**Taxon**	**PR2V9AL**	**A:SG_1.2**	**A:SG_4.3**	**A:SG_7.3**	**A:SG_11.8**	**A:SG_16.4**
Amoebozoa	132.3	0	0	3	0	3
Apicomplexa	118.7	116	195	3427	468	1005
Arthropoda	127.7	0	0	17	4	38
Ascomycota	123.4	8	2	3	2	5
Bacillariophyta	123.5	0	0	9	3	6
Cercozoa	124	0	4	41	27	24
Chordata	122.3	5	4	71	45	56
Ciliophora	108.6	1	16	103	29	43
Cnidaria	127.1	1	1	10	15	9
Dinophyceae	125.9	4	7	230	48	28
Foraminifera	120.2	9	22	204	88	175
Polycystinea	134.7	3	1	14	16	24
Rhizaria	126.2	1	8	121	33	104
Stramenopiles	122.3	14	13	206	105	115
Streptophyta	124.5	0	0	3	0	1
Pearson correlation coefficient *r*		-0.27269	-0.33233	-0.28064	-0.32559	-0.30078

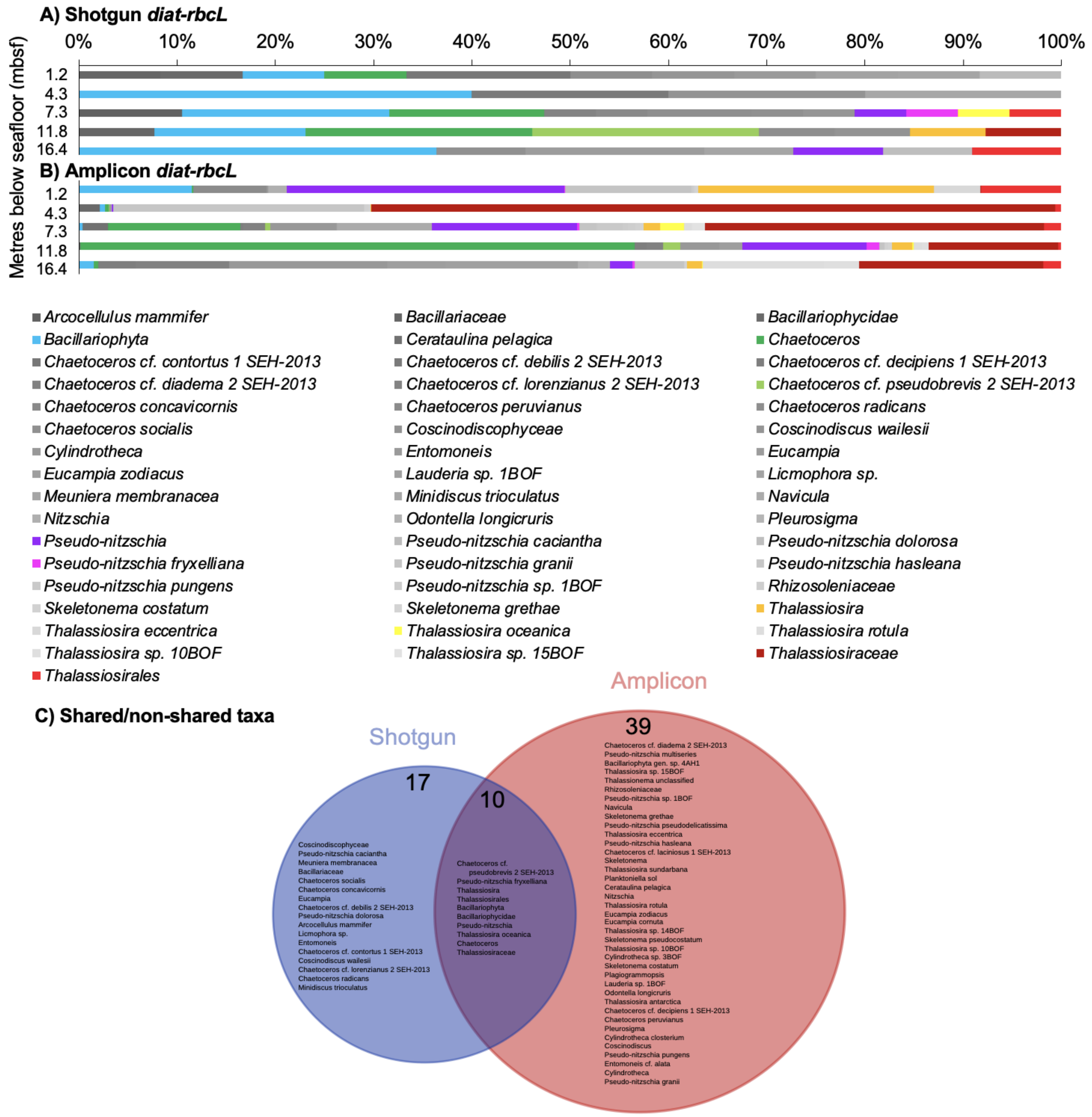

Supplementary Material Figure 2. Diatom relative abundance in shotgun and amplicon data after alignment with *diat-rbcL* database. Taxa are listed on species level, with A) and B) corresponding to Main Text Fig. 4, and C) listing the individual taxa names. See Supplementary Material Table 5 for list of taxa names.

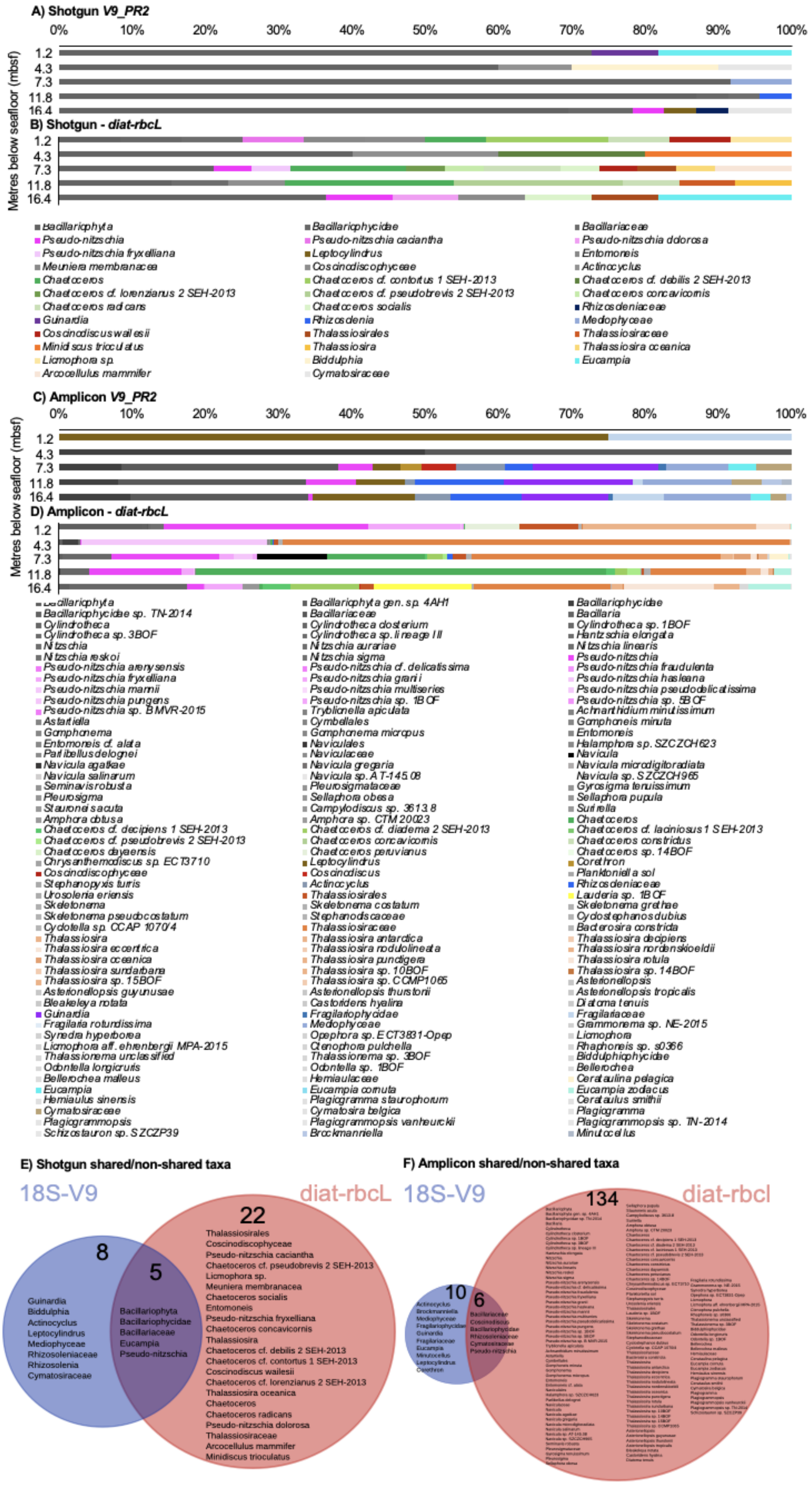

Supplementary Material Figure 3. Comparison of diatom relative abundance in shotgun and amplicon data after alignment with *V9_PR2* and *diat-rbcL* database. Taxa are listed on species level. See Supplementary Material Table 5 for list of species names and read counts.

Supplementary Material Table 5. Diatom read counts in shotgun and amplicon data after alignment with *V9_PR2* and *diat-rbcL* database. Shotgun diatom read counts data is shown in the first part of the Table, followed by amplicon diatom read counts (species level).

**Shotgun**	***V9_PR2***					***diat-rbcL***				
**Sample**	**Sample55-56**	**Sample41-42**	**Sample29-30**	**Sample19-20**	**Sample1-2**	**Sample55-56**	**Sample41-42**	**Sample29-30**	**Sample19-20**	**Sample1-2**
**mbsf**	**1.2**	**4.3**	**7.3**	**11.8**	**16.4**	**1.2**	**4.3**	**7.3**	**11.8**	**16.4**
Bacillariophyta	8	6	11	20	16	1	2	4	2	4
Bacillariophycidae	0	0	0	1	2	1	0	0	0	0
Bacillariaceae	0	0	0	1	0	1	0	0	1	0
Pseudo-nitzschia	0	0	0	0	1	0	0	1	0	1
Pseudo-nitzschia caciantha	0	0	0	0	0	1	0	0	0	0
Pseudo-nitzschia dolorosa	0	0	0	0	0	0	0	0	0	1
Pseudo-nitzschia fryxelliana	0	0	0	0	0	0	0	1	0	0
Leptocylindrus	0	0	0	0	1	0	0	0	0	0
Entomoneis	0	0	0	0	0	1	0	0	0	0
Meuniera membranacea	0	0	0	0	0	1	0	0	0	1
Coscinodiscophyceae	0	0	0	0	0	0	1	0	1	0
Actinocyclus	0	1	0	0	0	0	0	0	0	0
Chaetoceros	0	0	0	0	0	1	0	3	3	0
Chaetoceros cf. *contortus* 1 SEH-2013	0	0	0	0	0	2	0	0	0	0
Chaetoceros cf. *debilis* 2 SEH-2013	0	0	0	0	0	0	1	0	0	0
Chaetoceros cf. *lorenzianus* 2 SEH-2013	0	0	0	0	0	0	0	1	0	0
Chaetoceros cf. *pseudobrevis* 2 SEH-2013	0	0	0	0	0	0	0	0	3	0
Chaetoceros concavicornis	0	0	0	0	0	0	0	1	0	0
Chaetoceros radicans	0	0	0	0	0	1	0	2	1	0
Chaetoceros socialis	0	0	0	0	0	0	0	1	0	1
Rhizosoleniaceae	0	0	0	0	1	0	0	0	0	0
Guinardia	1	0	0	0	0	0	0	0	0	0
Rhizosolenia	0	0	0	1	0	0	0	0	0	0
Mediophyceae	0	0	1	0	0	0	0	0	0	0
Coscinodiscus wailesii	0	0	0	0	0	1	0	1	0	0
Thalassiosirales	0	0	0	0	0	0	0	1	0	1
Thalassiosiraceae	0	0	0	0	0	0	0	0	1	0
Minidiscus trioculatus	0	0	0	0	0	0	1	0	0	0
Thalassiosira	0	0	0	0	0	0	0	0	1	0
Thalassiosira oceanica	0	0	0	0	0	0	0	1	0	0
Licmophora sp.	0	0	0	0	0	1	0	0	0	0
Biddulphia	0	2	0	0	0	0	0	0	0	0
Eucampia	2	0	0	0	0	0	0	0	0	2
Arcocellulus mammifer	0	0	0	0	0	0	0	2	0	0
Cymatosiraceae	0	1	0	0	2	0	0	0	0	0

Amplicon	*V9_PR2*					*diat-rbcL*				
**Sample**	**Sample55-56**	**Sample41-42**	**Sample29-30**	**Sample19-20**	**Sample1-2**	**Sample55-56**	**Sample41-42**	**Sample29-30**	**Sample19-20**	**Sample1-2**
**mbsf**	**1.2**	**4.3**	**7.3**	**11.8**	**16.4**	**1.2**	**4.3**	**7.3**	**11.8**	**16.4**
Bacillariophyta	0	0	0	0	0	607	20	51	16	467
Bacillariophyta gen. sp. 4AH1	0	0	0	0	0	40	0	24	0	3
Bacillariophycidae	0	1	9	6	14	0	75	0	31	7
Bacillariophycidae sp. TN-2014	0	0	0	0	0	0	1	0	1	2
Bacillariaceae	0	1	31	19	35	6	0	0	0	0
Bacillaria	0	0	0	0	0	0	0	0	0	1
Cylindrotheca	0	0	0	0	0	8	5	922	1018	5096
Cylindrotheca closterium	0	0	0	0	0	12	0	14	8	20
Cylindrotheca sp. 1BOF	0	0	0	0	0	0	0	0	2	0
Cylindrotheca sp. 3BOF	0	0	0	0	0	0	0	0	1	11
Cylindrotheca sp. lineage III	0	0	0	0	0	0	0	0	0	6
Hantzschia elongata	0	0	0	0	0	0	1	0	0	0
Nitzschia	0	0	0	0	0	97	0	0	0	0
Nitzschia aurariae	0	0	0	0	0	0	0	1	0	0
Nitzschia linearis	0	0	0	0	0	0	1	0	0	0
Nitzschia reskoi	0	0	0	0	0	1	0	0	0	0
Nitzschia sigma	0	0	0	0	0	0	0	1	1	0
Pseudo-nitzschia	0	0	5	5	1	1493	6	2046	3247	733
Pseudo-nitzschia arenysensis	0	0	0	0	0	0	0	0	3	0
Pseudo-nitzschia cf. *delicatissima*	0	0	0	0	0	0	0	0	0	4
Pseudo-nitzschia fraudulenta	0	0	0	0	0	1	0	6	0	0
Pseudo-nitzschia fryxelliana	0	0	0	0	0	2	0	29	339	70
Pseudo-nitzschia granii	0	0	0	0	0	676	0	237	119	1580
Pseudo-nitzschia hasleana	0	0	0	0	0	6	901	0	10	0
Pseudo-nitzschia mannii	0	0	0	0	0	0	0	0	6	0
Pseudo-nitzschia multiseries	0	0	0	0	0	5	0	4	0	0
Pseudo-nitzschia pseudodelicatissima	0	0	0	0	0	0	0	3	18	8
Pseudo-nitzschia pungens	0	0	0	0	0	0	1	374	0	0
Pseudo-nitzschia sp. 1BOF	0	0	0	0	0	11	0	72	0	0
Pseudo-nitzschia sp. 5BOF	0	0	0	0	0	0	0	1	0	0
Pseudo-nitzschia sp. B MVR-2015	0	0	0	0	0	0	0	0	1	5
Tryblionella apiculata	0	0	0	0	0	0	0	1	0	0
Achnanthidium minutissimum	0	0	0	0	0	0	0	0	1	1
Astartiella	0	0	0	0	0	0	0	0	1	0
Cymbellales	0	0	0	0	0	0	0	1	0	0
Gomphoneis minuta	0	0	0	0	0	0	0	0	0	2
Gomphonema	0	0	0	0	0	0	0	0	1	0
Gomphonema micropus	0	0	0	0	0	0	0	0	0	1
Entomoneis	0	0	0	0	0	0	1	0	2	0
Entomoneis cf. *alata*	0	0	0	0	0	0	10	0	2	0
Naviculales	0	0	0	0	0	0	1	0	1	0
Halamphora sp. SZCZCH623	0	0	0	0	0	1	0	0	0	0
Parlibellus delognei	0	0	0	0	0	0	0	0	5	0
Naviculaceae	0	0	0	0	0	0	0	1	0	1
Navicula	0	0	0	0	0	1	1	1324	0	5
Navicula agatkae	0	0	0	0	0	0	0	0	0	1
Navicula gregaria	0	0	0	0	0	0	0	3	0	0
Navicula microdigitoradiata	0	0	0	0	0	0	0	0	1	0
Navicula salinarum	0	0	0	0	0	0	0	1	0	1
Navicula sp. AT-145.08	0	0	0	0	0	0	0	0	0	1
Navicula sp. SZCZCH965	0	0	0	0	0	0	0	0	1	0
Seminavis robusta	0	0	0	0	0	0	0	1	0	0
Pleurosigmataceae	0	0	0	0	0	0	0	0	0	1
Gyrosigma tenuissimum	0	0	0	0	0	0	0	0	0	1
Pleurosigma	0	0	0	0	0	1	1	0	0	695
Sellaphora obesa	0	0	0	0	0	0	0	0	0	2
Sellaphora pupula	0	0	0	0	0	0	0	0	0	1
Stauroneis acuta	0	0	0	0	0	0	0	1	0	0
Campylodiscus sp. 3613.8	0	0	0	0	0	0	0	0	0	1
Surirella	0	0	0	0	0	0	0	0	0	2
Amphora obtusa	0	0	0	0	0	0	0	0	0	2
Amphora sp. CTM 20023	0	0	0	0	0	0	0	1	0	3
Chaetoceros	0	0	0	0	0	9	12	1865	14494	144
Chaetoceros cf. *decipiens* 1 SEH-2013	0	0	0	0	0	0	0	40	306	1207
Chaetoceros cf. *diadema* 2 SEH-2013	0	0	0	0	0	0	0	308	442	3021
Chaetoceros cf. *laciniosus* 1 SEH-2013	0	0	0	0	0	0	0	0	0	12
Chaetoceros cf. *pseudobrevis* 2 SEH-2013	0	0	0	0	0	0	3	72	447	2
Chaetoceros concavicornis	0	0	0	0	0	0	0	0	1	0
Chaetoceros constrictus	0	0	0	0	0	0	0	4	0	0
Chaetoceros dayaensis	0	0	0	0	0	0	0	3	0	0
Chaetoceros peruvianus	0	0	0	0	0	393	0	0	3	0
Chaetoceros sp. 14BOF	0	0	0	0	0	0	0	0	0	4
Chrysanthemodiscus sp. ECT3710	0	0	0	0	0	0	0	1	0	0
Leptocylindrus	3	0	4	5	20	0	0	0	0	0
Corethron	0	0	3	0	0	0	0	0	0	0
Coscinodiscophyceae	0	0	0	0	0	0	0	1	0	0
Coscinodiscus	0	0	5	0	0	0	0	0	13	9

Planktoniella sol	0	0	0	0	0	0	0	0	26	0
Stephanopyxis turris	0	0	0	0	0	0	0	2	0	0
Actinocyclus	0	0	7	1	7	0	0	0	0	0
Rhizosoleniaceae	0	0	4	9	14	0	2	104	0	0
Urosolenia eriensis	0	0	0	0	0	0	0	0	1	0
Thalassiosirales	0	0	0	0	0	434	23	245	96	579
Lauderia sp. 1BOF	0	0	0	0	0	0	0	0	2	4290
Skeletonema	0	0	0	0	0	2	0	2	6	1
Skeletonema costatum	0	0	0	0	0	8	14	59	67	63
Skeletonema grethae	0	0	0	0	0	16	8	49	125	33
Skeletonema pseudocostatum	0	0	0	0	0	0	0	1	2	6
Stephanodiscaceae	0	0	0	0	0	2	0	0	0	0
Cyclostephanos dubius	0	0	0	0	0	0	0	0	2	0
Cyclotella sp. CCAP 1070/4	0	0	0	0	0	0	0	0	1	0
Thalassiosiraceae	0	0	0	0	0	3	2458	4758	3384	5965
Bacterosira constricta	0	0	0	0	0	0	0	0	0	3
Thalassiosira	0	0	0	0	0	1269	2	237	532	492
Thalassiosira antarctica	0	0	0	0	0	1	0	0	1	15
Thalassiosira decipiens	0	0	0	0	0	0	0	2	0	1
Thalassiosira eccentrica	0	0	0	0	0	248	0	0	3	0
Thalassiosira nodulolineata	0	0	0	0	0	6	0	1	0	0
Thalassiosira nordenskioeldii	0	0	0	0	0	0	0	0	0	1
Thalassiosira oceanica	0	0	0	0	0	0	0	338	31	26
Thalassiosira punctigera	0	0	0	0	0	0	0	0	1	0
Thalassiosira rotula	0	0	0	0	0	0	0	100	226	3923
Thalassiosira sundarbana	0	0	0	0	0	0	0	8	1	21
Thalassiosira sp. 10BOF	0	0	0	0	0	0	1	16	160	1118
Thalassiosira sp. 14BOF	0	0	0	0	0	0	0	12	17	3
Thalassiosira sp. 15BOF	0	0	0	0	0	0	0	173	2	0
Thalassiosira sp. CCMP1065	0	0	0	0	0	0	0	0	2	4
Asterionellopsis	0	0	0	0	0	0	1	0	0	0
Asterionellopsis guyunusae	0	0	0	0	0	0	0	0	2	0
Asterionellopsis thurstonii	0	0	0	0	0	0	0	0	1	1
Asterionellopsis tropicalis	0	0	0	0	0	0	0	0	1	0
Bleakeleya notata	0	0	0	0	0	0	0	1	0	6
Castoridens hyalina	0	0	0	0	0	0	0	2	0	1
Diatoma tenuis	0	0	0	0	0	0	1	0	0	0
Guinardia	0	0	18	13	17	0	0	0	0	0
Fragilariophycidae	0	0	1	0	1	0	0	0	0	0
Fragilariaceae	1	0	0	1	10	0	0	0	0	0
Fragilaria rotundissima	0	0	0	0	0	1	0	0	0	0
Mediophyceae	0	0	9	9	17	0	0	0	0	0
Grammonema sp. NE-2015	0	0	0	0	0	0	2	2	1	0
Synedra hyperborea	0	0	0	0	0	0	0	1	0	1
Opephora sp. ECT3831-Opep	0	0	0	0	0	0	2	0	0	1
Licmophora	0	0	0	0	0	0	0	1	0	0
Licmophora aff. *ehrenbergii* MPA-2015	0	0	0	0	0	0	0	2	0	0
Ctenophora pulchella	0	0	0	0	0	0	0	1	3	1
Rhaphoneis sp. s0366	0	0	0	0	0	0	0	0	0	1
Thalassionema unclassified	0	0	0	0	0	0	0	28	0	0
Thalassionema sp. 3BOF	0	0	0	0	0	0	0	2	0	0
Biddulphiophycidae	0	0	0	0	0	0	0	1	0	1
Odontella longicruris	0	0	0	0	0	0	0	0	1	339
Odontella sp. 1BOF	0	0	0	0	0	3	0	0	0	0
Bellerochea	0	0	0	0	0	0	0	0	0	1
Bellerochea malleus	0	0	0	0	0	0	0	0	0	1
Hemiaulaceae	0	0	0	0	0	0	0	6	0	0
Cerataulina pelagica	0	0	0	0	0	1	0	353	1	6
Eucampia	0	0	4	0	4	0	0	0	0	0
Eucampia cornuta	0	0	0	0	0	0	0	13	1	2
Eucampia zodiacus	0	0	0	0	0	1	0	15	606	1879
Hemiaulus sinensis	0	0	0	0	0	0	0	1	0	0
Plagiogramma staurophorum	0	0	0	0	0	0	0	0	5	0
Cerataulus smithii	0	0	0	0	0	0	0	0	0	3
Cymatosiraceae	0	0	5	3	3	0	0	2	0	0
Cymatosira belgica	0	0	0	0	0	0	0	3	1	0
Plagiogramma	0	0	0	0	0	0	0	0	0	2
Plagiogrammopsis	0	0	0	0	0	0	0	22	0	0
Plagiogrammopsis vanheurckii	0	0	0	0	0	0	0	0	0	1
Plagiogrammopsis sp. TN-2014	0	0	0	0	0	0	0	1	0	0
Schizostauron sp. SZCZP39	0	0	0	0	0	0	0	1	0	0
Brockmanniella	0	0	0	2	1	0	0	0	0	0
Minutocellus	0	0	0	1	0	0	0	0	0	0

References:

Clarke EL, Taylor LJ, Zhao C, Connell A, Lee JJ, Fett B, Bushman FD, Bittinger K. Sunbeam: An extensible pipeline for analyzing metagenomic sequencing experiments. *Microbiome* 2019; 7, 46.

Ewels P, Magnusson M, Lundin S, Käller M. MultiQC: summarize analysis results for multiple tools and samples in a single report. *Bioinformatics* 2016; 32, 3047-3048.

Herbig A, Maixner F, Bos KI, Zink A, Krause J, Huson DH. malt: Fast alignment and analysis of metagenomic DNA sequence data applied to the Tyrolean Iceman. *BioRxiv* 2016, 050559.

Huson DH, Beier S, Flade I, Górska A, El-Hadidi M, Mitra S, Tappu R. MEGAN Community Edition - Interactive exploration and analysis of large-scale microbiome sequencing data. *PLOS Computational Biology* 2016; 12, e1004957.

Li W, Godzik A. Cd-hit: a fast program for clustering and comparing large sets of protein or nucleotide sequences. *Bioinformatics* 2006; 22, 1658-1659.

Schubert M, Lindgreen S, Orlando L. adapterremoval v2: Rapid adapter trimming, identification, and read merging. *BMC Research Notes* 2016; 9, 88.
